# In-Depth Tanscriptomic Analysis on Giant Freshwater Prawns

**DOI:** 10.1371/journal.pone.0060839

**Published:** 2013-05-29

**Authors:** Maizatul Izzah Mohd-Shamsudin, Yi Kang, Zhao Lili, Tian Tian Tan, Qi Bin Kwong, Hang Liu, Guojie Zhang, Rofina Yasmin Othman, Subha Bhassu

**Affiliations:** 1 Genomics and Evolutionary Biology Lab, Centre for Research in Biotechnology for Agriculture (CEBAR) and Institute of Biological Sciences, Faculty of Science, University of Malaya, Kuala Lumpur, Malaysia; 2 Beijing Genomics Institute, Shenzhen, China; Auburn University, United States of America

## Abstract

Gene discovery in the Malaysian giant freshwater prawn (*Macrobrachium rosenbergii*) has been limited to small scale data collection, despite great interest in various research fields related to the commercial significance of this species. Next generation sequencing technologies that have been developed recently and enabled whole transcriptome sequencing (RNA-seq), have allowed generation of large scale functional genomics data sets in a shorter time than was previously possible. Using this technology, transcriptome sequencing of three tissue types: hepatopancreas, gill and muscle, has been undertaken to generate functional genomics data for *M. rosenbergii* at a massive scale. *De novo* assembly of 75-bp paired end Ilumina reads has generated 102,230 unigenes. Sequence homology search and *in silico* prediction have identified known and novel protein coding candidate genes (∼24%), non-coding RNA, and repetitive elements in the transcriptome. Potential markers consisting of simple sequence repeats associated with known protein coding genes have been successfully identified. Using KEGG pathway enrichment, differentially expressed genes in different tissues were systematically represented. The functions of gill and hepatopancreas in the context of neuroactive regulation, metabolism, reproduction, environmental stress and disease responses are described and support relevant experimental studies conducted previously in *M. rosenbergii* and other crustaceans. This large scale gene discovery represents the most extensive transcriptome data for freshwater prawn. Comparison with model organisms has paved the path to address the possible conserved biological entities shared between vertebrates and crustaceans. The functional genomics resources generated from this study provide the basis for constructing hypotheses for future molecular research in the freshwater shrimp.

## Background

The Malaysian giant freshwater prawns, scientifically known as *Macrobrachium rosenbergii* (de Man, 1879), is the largest Palaemonid shrimp of one of the most diverse genera for freshwater Crustacea [1], [2]. It is commonly found along coastal river systems: it lives in the freshwater as an adult but the larvae require a brackish environment for survival and development [3], [4]. This discovery had led to the development of mass rearing techniques for commercial production [5]. Since then, this species has been widely cultured both in and outside its native range and for human consumption. Annual revenue generated from its commercial production has been estimated at approximately one billon USD per annum (FAO, 2002). Its commercial significance has thus spawned interest on research addressing various aspects that are directly or indirectly affecting commercial production: fisheries, nutrition, reproduction and bacterial and viral disease as well as environmental stress response.

Despite great interest in this organism, gene discovery in *M. rosenbergii* has been conducted at a relatively small scale compared to another freshwater prawns of the same genus, and also compared with other economically important shrimp including the oriental river prawn (*M.* nipponense), the marine Penaeid shrimps (*Litopenaeus vannamei, Penaeus monodon, Marsupenaeus japonicus* and *Fenneropenaeus chinensis)* and other marine Malacostracas (Petrolisthes cinctipes, Homarus americanus). As compared to the currently available 11 expressed sequence tags (ESTs) of *M. rosenbergii* in the public EST database (accessed on June 2, 2010), the EST sequencing of the oriental river prawn, *M. nipponense*, ovary cDNA library has already generated 3,256 ESTs, thus representing the largest addition to the freshwater shrimp EST data [6], far exceeding EST information available for *M. rosenbergii*. Gene discovery through EST sequencing has also been conducted on more than nine tissue types and various physiological conditions for marine *Penaeid* shrimps to identify immune-related, reproduction-related, sex-related and differentially expressed genes between normal and diseased individuals [7]. In addition, the third largest EST sequencing project for crustaceans is currently represented by transcriptome characterization of the porcelain crab, which generated approximately 100,000 high quality ESTs [8], providing an even greater amount of sequence information for genome level comparative study among the Malacostracas in the crustacean taxa.

Next generation sequencing (NGS) technologies are revolutionizing genomic research with their ability to provide enormous amounts of sequence data with a greater breadth and depth of information in shorter times and at a significantly lower cost than the traditional Sanger sequencing technology. Recently, NGS technology has been used to sequence and characterize the whole transcriptome of organisms in various organs and cell lines [9], [10], [11]. RNA-seq's ability to provide both qualitative and quantitative information of gene expression at higher sensitivity and accuracy has given this technology a primary advantage over precedent transcriptomic methods such as EST sequencing, serial analysis of gene expression (SAGE), massively parallel signature sequencing (MPSS) and microarrays. Furthermore, RNA-seq does not require any in-depth understanding of the genome where the transcriptome is derived from, which makes it the method of choice for rapid characterization of the entire transcriptome of non-model organisms [11].

Taking advantage of the RNA-seq technology, the transcriptome of three main organs in *M. rosenbergii* were sequenced: gills, hepatopancreas and muscle to (i) generate large amounts of EST sequence data for gene discovery, (ii) mine simple sequence repeat markers that represent a resource for trait mapping and (iii) generate differential gene expression profiles of these main organs to better understand their functions in the giant freshwater prawn. The data obtained from this study can be further utilized to develop specific DNA/RNA markers that can be used in future molecular studies to address a wide range of specific research questions.

## Materials and Methods

### Total RNA extraction

Adult prawns were obtained from a hatchery at Jelebu, Negeri Sembilan, Malaysia. Gill and hepatopancreas tissues were collected from a pool of individuals that are full sibs from a breeding program which are stocked in the same pond reducing environment effectswhile muscle tissues were obtained from two individuals (one normal and one infected individual) which had been screened for infectious hypodermal and hematopoietic necrosis virus (IHHNV) infection using 389F/389R OIE primers sets [12]. The tissues were immediately frozen in liquid nitrogen after dissection before storage at −80^ o^C until RNA extraction. Total RNA (∼20 µg) was extracted from the tissues using Trizol reagent (Invitrogen, Carlsband, CA, USA) following the manufacturers' protocols before dissolving it in diethylpyrocarbonate (DEPC) treated water to give a final concentration of >750ng/µL. The dissolved total RNA was stored at −80^ o^C prior to RNA sequencing.

### Illumina sequencing

The cDNA libraries preparation and sequencing were conducted using Illumina sequencing technology (Beijing Genome Institute, Shenzhen, China). Poly A+ mRNA was isolated from the total RNA using Sera-mag Magnetic Oligo (dT) Beads (Illumina). The extracted RNA was hydrolysed to smaller fragments (∼150–200 bp) to avoid priming bias. Double stranded cDNA was synthesized from the fractionated RNA pool using reverse transcriptase, RNase H (Invitrogen) and DNA polymerase I (New England BioLabs) primed with random hexamers. Four sequencing cDNA libraries of gills, hepatopancrease, muscle (Infected and non- infected) were prepared according to the manufacturer's protocols (Illumina) before being subjected to 75 bp paired end sequencing using an Illumina Genome Analyzer platform.

### *De novo* assembly and assessment

After filtering out any contamination and low quality reads, the clean reads were clipped into 25-bp kmers, assembled into contigs and joined into scaffolds based on the reads' paired-end information using the SOAPdenovo software [13]. To avoid redundancy, the contigs and scaffolds from each library were subjected to clustering using TGI Clustering tool [14] to generate library-specific unigenes. One set of standard unigene sequences was generated by clustering library-specific unigenes from all four libraries. For assembly quality assessment, the resultant unigenes were BLASTed [15] against *M. rosenbergii* assembled EST and mRNA sequences obtained from the NCBI EST and nucleotide databases.

### Protein coding gene identification and classification

The unigenes sequences were annotated by using homology search (BLASTX) [15] with E-value cutoff of 10^−5^ against NCBI non-redundant (Nr) database, Swissprot, Cluster of Orthologous Groups database (COG) [16], [17] and Kyoto Encyclopedia of Genes and Genome (KEGG) database [18]. Gene Ontology [19] assignment was conducted using BLAST2GO software [20] with default parameters. The coding sequence and the gene direction of the annotated unigenes were determined based on the BLAST results from four databases aforementioned. For those sequences that had no annotation, the coding sequence and direction were predicted using ESTscan [21] using BLAST predicted coding sequence data as the training set.

### Non-coding RNA identification and characterization

To identify the non-coding RNA in the unigenes, the remaining unigenes that had no BLAST protein annotation and no coding sequence predicted using both EMBOSS getORF and ESTscan were subjected to BLAST (E-value < = 0.01) sequence homology search against the Rfam 10.0 database [22], [23]. RNA structure and sequence similarity search (INFERNAL program) using covariance model (CMsearch) [24] was conducted on the significant hits from the BLAST search results.

### Repetitive elements and simple sequence repeat (SSR) markers mining

Repetitive elements in the whole transcriptome were identified using RepeatMasker 3.2.9 [25] with the species option set as Arthropoda. Simple sequence repeats (SSR) or microsatellites identified were further characterized for their distribution across the transcriptome.

SSR loci selection for primer design was carried out using the Window based iQDD software [26]. Microsatellite was set as minimum five repetitions of 2–6 bp motifs. ‘All against all’ BLAST (E-value cutoff: 10^−40^) [15] was performed on sequences with soft-masked microsatellites. Only the unique sequences which had no BLAST hit to other unigenes and no short repeats in the flanking regions were selected for the PCR primer design (default parameters, minimum PCR product size  = 150).

Thirty five primer sets amplifying SSR loci associated with known protein coding genes were selected for further testing. They were tested for successful SSR amplification and size verification of expected PCR products in 16 individuals. Each PCR amplification was performed in a total volume of 10 µl of PCR mixture consisting of 1.2 µl of distilled water, 3.0 µl of 5X Green GoTaq PCR Buffer (Promega), 1.5 µl of MgCl_2_ (25 mM), 0.25 µl of each dATP, dGTP, dCTP and dTTP, 0.5µl of each forward primer and reverse primer (10 µM), 2.0 µl (20ng) of DNA from each sample and 0.3 µl Promega GoTaq® Polymerase (5 U/µl). The Bio-Rad C1000 Thermal Cycling profile was as follows: 3 min of initial denaturation at 96°C, 39 cycles of denaturation at 94°C for 10 s, annealing for 45 sec (shown in Table 1 for primers with polymorphic loci only) and extension at 72°C for 30 sec. The final extension at 72°C for 7 min was performed before it was maintained at 4°C. PCR products were run on 1.0% agarose gels stained with ethidium bromide to test for successful amplification and on 4.0% Metaphor® agarose gel for the first polymorphism screening. Subsequent polymorphism genotyping using ABI 3130 genetic analyzer (ABI, Inc.) will be performed on four populations of cultured prawns, and the results described in a separate publication.

**Table 1:** Primers and potential polymorphic status for 23 successfully amplified SSR.

LocusID	GeneID	Motif	Primer_sequence F: forward; R: reverse	Tm	PCR ProductSize	Potential Polymorphic
MR5	Unigene7776_All	(TC)_6_	F: TTCCCCAATGCTTCTTCATC R: ACGCACCTCCTTGTATCCAC	55.0	150–177	YES
MR7	Unigene4546_All	(CT)_6_	F: GTTTCCGATGCCAAAGACAT R: ATATCGACGGCCTCTTGGTA	63.3	150–170	NULL
MR8	Unigene9496_All	(TC)_6_	F: ACTTCTTGGCTTCAAGGGCT R: TCCAGTCAAAAGAATTCGCA	55.0	150–200	YES
MR13	Unigene26276_All	(TC)_7_	F: TGGACATCTTTGCATAGCCA R: CACATCGGGGTTATTTTGGT	61.4	150–160	YES
MR14	Unigene12899_All	(CT)_8_	F: CTCTGCTTCGTAAAATCGCC R: GAACACTTTTGGCATGGGAG	61.4	150–165	YES
MR18	Unigene54607_All	(TC)_10_	F: GTCCTCACAGCTGGTTCGAT R: ATTTAACCCCTCGCCATTCT	65.0	250–286	YES
MR20	Unigene9630_All	(TG)_13_	F: GAACCGCATACATTTTCCCC R: GAGAAATTTGGACATTGTCGG	63.3	150–150	NULL
MR23	Unigene51169_All	(TC)_23_	F: CAAAGTGAGATTCATTACGGAGG R: GCCTTCATTTGGCATTGAAA	55.0	150–152	NO
MR24	Unigene2731_All	(AGC)_5_	F: ACTCCACCAAACATTGAGCC R: CCCCTCAAGTGGTCAGTCAT	61.4	150–180	NULL
MR31	Unigene16282_All	(TTG)_5_	F: CGCCCAAGATCTGATCCAC R: ATCTCAACAGTAACATGGACTCAAAC	64.5	150–150	NO
MR32	Unigene10567_All	(ACC)_5_	F: AATCGATCATCACCAGCCTC R: TTGTTCCAACAGAACCCTCC	64.5	150–184	YES
MR39	Unigene7685_All	(ATT)_6_	F: GGTGGACTGAGACTGGCTTC R: TGACAATGCAGATTCCCAAA	64.5	250–294	NO
MR40	Unigene18380_All	(TCC)_6_	F: TCAGAGATGTATTCCCCACAGA R: TCCCCTGATCTTTAAATCCTCC	64.5	150–150	NO
MR41	Unigene11347_All	(TCA)_7_	F: TCTCGTGTGACATAGGCAGC R: TTGGAAGCAGAGAACAAGATTTC	63.3	150–199	YES
MR47	Unigene9912_All	(GGA)_7_	F: AAGTGGAGGTGGAACAGGTG R: CTGAGACGGTCTTCTCCCTG	60.0	300–302	YES
MR51	Unigene826_All	(CTT)_7_	F: AGCTGTACACCTCTGGCTCG R: CTACGAAACGCATGGTTGG	63.3	150–150	YES
MR52	Unigene9209_All	(TCC)_7_	F: CCACTCCATTCACTCCCACT R: CTACACCACCACAGACACCG	63.3	200–228	YES
MR53	Unigene33994_All	(CAT)_7_	F: TGGATGTATAACTACGACGACAGC R: CCGACTCTTCTTCGTCTTCC	61.4	150–159	YES
MR55	Unigene35088_All	(CCT)_6_	F: GTCCGAGTGGCCTAGGGT R: TTGGAATCCAGCTCTGAAGG	65.0	150–163	YES
MR56	Unigene9209_All	(CCT)_9_	F: GAGACAAGCCGTGAAGGAAG R: AGTGAATGGAGTGGGTGGAG	61.4	150–183	YES
MR57	Unigene33873_All	(AGG)_13_	F: CGCTGTGCTGTACATGACCT R: TGGTGTTAGGAACAATGTCG	65.0	150–150	NULL
MR58	Unigene51169_All	(TCT)_24_	F: AGTCTCCTAAGACCCCGGAA R: TATCGTCGCCATCACTAGCA	65.0	300–301	YES
MR59	Unigene1832_All	(ATTT)_5_	F: TTTCATGGAAATTTGGCACA R: TGGTCTGAGAAGTGTTTTATTTCA	65.0	200–204	NULL

### Expression analysis and normalization

Clean reads from each library were mapped to the standard unigene set using SOAP [27] with a maximum of two mismatches allowed. For paired end reads mapping, an insert size of range less than ∼60 bp was allowed, depending on the size of the fragments during the RNA fragmentation for each sample. The expression level based on the number of reads mapped to the standard unigenes was normalized by the number of reads per kilobase per million reads (RPKM) [9].
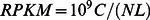
(1)Where *C* is the number of mappable reads that fell onto the unigene, *N* is the total number of mappable reads in the experiment and *L* is the total length of the unigene in base pair.

### Differential gene expression analysis and functional enrichment

The significance of differentially expressed genes (DGE) was evaluated by using following statistical tests:

- Statistical algorithm developed by Audic and Claverie (1997) [28]

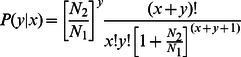
(2)Where N1 are total clean reads from sample 1, N2 are total clean reads from sample 2, x are number of reads from sample 1 mapped to unigene and y are number of reads from sample 2 mapped to unigene.
- False discovery rate analysis for multiple testing (FDR; cutoff value: 0.001) [29]
- The absolute value of the unigene RPKM ratio in two samples (cutoff: 1):

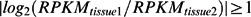
(3)


GO functional and KEGG pathway enrichment of the DGE were performed and statistically tested with a hypergeometric test:
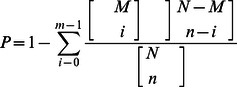
(4)Whereby *N* is the number of all unigenes with GO annotation, *n* is the number of DGE in *N*, *M* is number of all unigenes that are annotated to a certain GO term and *m* is the number of DGE in *M*.

P-values of GO functional enrichment were re-evaluated using Bonferroni correction while FDR was calculated for KEGG pathway enrichment. The significant cutoff value for the corrected p-value and FDR was set as 0.05 for both enrichments.

## Results and Discussion

### Sequencing, assembly, and clustering output

In total, 86 million clean 75 bp-paired-end reads from four *M. rosenbergii* tissue libraries, generated a total of 12.9 gigabases (Gb) of sequences. High quality sequencing data was reflected by the average Q_20_ of 92.50% and a percentage of unknown nucleotide (N percentage) of 0.05% for the four libraries.

**Figure 1:**
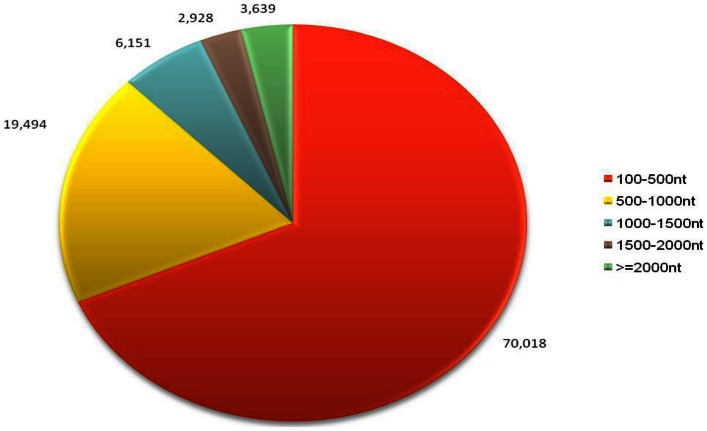
Size distribution of the assembled 102,230 unigenes. The abundance of unigenes assembled from four libraries: gill, hepatopancreas, healthy muscle and infected muscle based on nucleotide length (nt) in the size range of 100–500, 500–1,000, 1,000–1,500, 1500–2,000 and more than 2,000 nt.

*De novo* assembly of each library's clean raw reads generated a total of 595,195 contigs with size range of 75–500 bp. (Gills: 183,382; Hepatopancreas: 258,050; Normal Muscle: 84,530; Infected Muscle: 68,783). The contigs were assembled into 367,251 scaffolds of mean size 323 bp and average N_50_ of 425 bp. Clustering the scaffolds of each library resulted in a total of 177,275 library-specific unigenes. Clustering the library-specific unigenes from all four libraries resulted in 102,230 standard unigenes, with mean size of 566 bp and N_50_ of 746 bp. The length distribution of the unigenes obtained is illustrated in Figure 1. The reads data can be obtained from the NCBI Short Read Archive (SRA) under the accession number: SRA 045433.1, while the assembled sequence can be accessed in the NCBI Transcriptome Shotgun Assembly (TSA) Sequence archive under the accession number: TSA JP351514-JP355722. Assembly assessment of the unigenes had been shown to match *M. rosenbergii* publicly available EST and full mRNA sequence with E-value <10^−25^ (data not shown). Multiple unigenes matched to the same or different region of one gene can be attributed mainly to occurrence of alternative splicing, isoforms and incomplete coverage of the gene by one unigene.

### Identification of known and novel protein coding genes

BLASTX search of the unigenes against the NCBI nr, Swissprot, KEGG and COG databases returned 24,476 (23.94%), 20,261 (19.81%), 16,462 (16.10%) and 8,837 (8.64%) strong matches respectively, giving a final total of 24,739 unigenes (24.19%) annotated (Table S1 in File S1). The high number of non-annotated genes (∼75%) could be attributed to the lack of well annotated protein-coding genes nor any well characterized genome of *M. rosenbergii* or other Crustaceans in the public databases, as well as the large number of sequences (∼70%) with short lengths of 100–500 bp (Figure 1). From the BLAST annotated unigenes, only 24,500 unigenes had coding sequence longer than 300 bp. The resultant coding sequence data can be used as the training set for the prediction of novel protein coding genes.

Based on the ESTScan result on the unigenes with no matches from the BLAST search, 7,595 unigenes (7.43%) were predicted to contain coding sequence longer than 300 bp and thus classified as novel protein coding genes (Table S1 in File S2). All together, 32,334 unigenes (31.63%) were classified as protein coding genes in the transcriptome. The remaining unigenes were expected to consist of either messenger like non-coding RNA or fragments of untranslated region of protein coding genes.

### Classification of known protein coding genes

**Figure 2:**
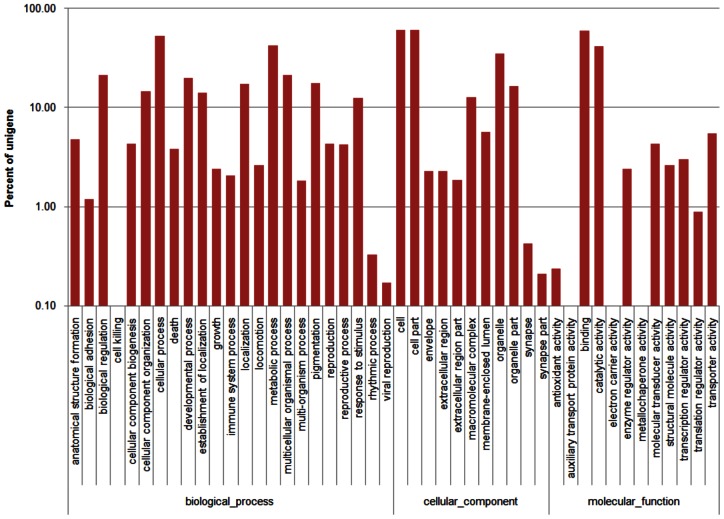
Gene Ontology classification of the 7,533 protein annotated unigenes. Unigene sequences were systematically classified into GO sub-categories under the Biological Process, Cellular Component and Molecular Function Gene Ontology Catalogue system. Each bar represents the relative abundance of unigenes classified under each sub-category.

Based on the protein annotation results from the Nr database homology search, 7,533 unigenes (Biological process: 5,231 unigenes; Cellular component: 4,689 unigenes; Molecular function: 6198 unigenes) could be assigned to 46 GO groups where the distribution of unigenes across all groups is presented in Figure 2 (Table S1 in File S1). Highly represented biological process ontology were biological regulation, cellular, metabolic, multicellular organismal and developmental processes, where each ontology was represented by more than 1,500 unigenes. GO assigned unigenes were mainly compartmentalized in the cell, organelle and macromolecular complex components. From the molecular function ontology analysis, a significantly large number of unigenes belonged to binding (4,490 unigenes) and catalytic activity (3,094 unigenes) categories. Together, the GO assignments for all three main categories were designated as representation of the general gene expression profile for the three tissues: hepatopancreas, gills and muscle of *M. rosenbergii* for the subsequent expression analysis.

**Figure 3:**
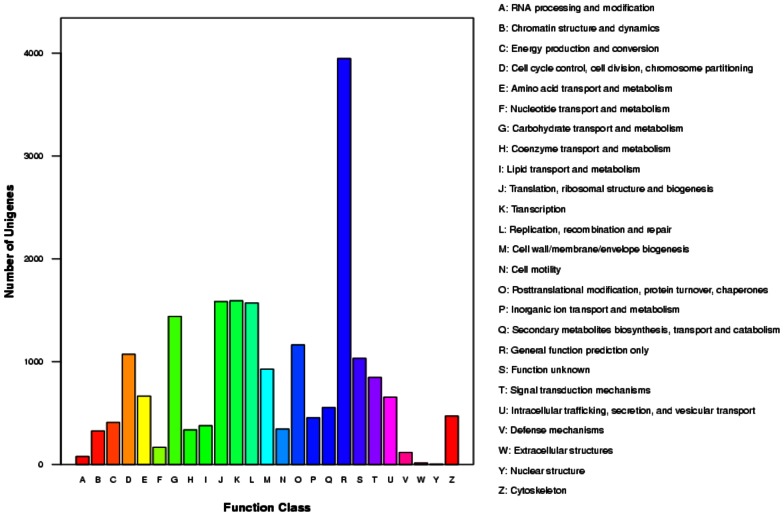
Histogram presentation of Clusters of Orthologus Groups classification of 8,837 known protein annotated unigenes. Each bar represents the number of unigenes classified into each of the 26 COG functional categories.

As shown in Figure 3, COG classification based on the BLAST search against the COG databases resulted in 8,837 unigenes categorized into 26 categories with the highest number of unigenes falling under general function prediction (3,947, 44.6%), followed by genetic information processing categories (transcription: 1,593, 17.9%; translation, ribosomal structure and biogenesis: 1,583, 17.7%; replication, recombination and repair: 1,569, 17.8%) and carbohydrate transport and metabolism (1,440, 16.3%). The functional categories ‘RNA processing and modification’, ‘extracellular’ and ‘nuclear’ structures had the least representation (<1% for each category) in the whole transcriptome data.

**Figure 4:**
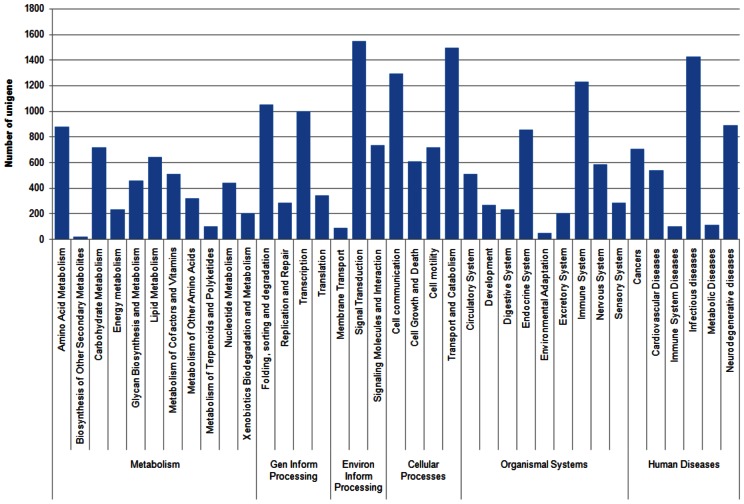
KEGG biological pathway classification histograms for 16, 403 protein annotated unigenes. Each bar represents the number of unigenes that are systematically categorized into sub-classes under Metabolism, Genetic Information Processing, Cellular Processes, Organismal Systems and Human Diseases.

Systematic classification of the unigenes based on KEGG biological pathway showed 16,403 unigenes being mapped to 218 KEGG pathways. At the highest categorical level, the transcriptome data covered 127 metabolic pathways, 87 human diseases pathways, 40 organismal system pathways, 19 environmental processing pathways, 17 genetic information processing pathways, and 13 cellular process pathways. The distribution of unigenes across the second categorical level of KEGG pathways is as shown in Figure 4. At the most specific categorical level, 2,342 unigenes (14.3%) were mapped to the global metabolic pathways and as high as 776 (4.73%) and 717 (4.37%) unigenes were mapped to spliceosome' pathway under ‘transcription’ category and ‘regulation of actin cytoskeleton' pathway under ‘cell motility’ category respectively. In addition, ‘pathways in cancer’, ‘focal adhesion’, ‘amoebiasis’ and ‘endocytosis’ are among the highest represented pathways (>3% of unigenes for each pathway) in the transcriptome data. The least represented pathways with less than 10 unigenes categorized in each pathways were ‘asthma’, ‘phenylalanine, tyrosine and tryptophan biosynthesis’, ‘D-glutamine and D-glutamate metabolism’, ‘biotin metabolism’, ‘vitamin B6 metabolism’, ‘lipoic acid metabolism’, ‘butirosin and neomycin biosynthesis’ and ‘polyketide sugar unit biosynthesis'.

Massive amounts of transciptome data have allowed for the revival of systems biology that aims to elucidate the function of a cell or more complex biological organization by conceptualizing interacting genes as systems or functional groups. Emergence of databases such as COG, GO and KEGG that aim to identify correlated genes for specific physiological processes and allow comparison and description of functional features using common terminologies have facilitated the extraction of biologically meaningful information from high throughput functional genomics data. However, the comprehensiveness of the databases poses a limitation or bias to the representation of overall functional information derived from each database. From the transcriptome data in this study, differences were observed in the distribution of molecular data representing a specific biological entity in the GO, COG and KEGG classifications (Figure 2–4) which can be attributed to differences in the coverage of protein coding genes by each classification system (GO: ∼23%; COG: ∼27%; KEGG: ∼50%). These differences may return different outcomes for the downstream analysis (e.g. functional and pathway enrichment) which utilize the information from these functional classifications.

### Identification of non-coding RNA

Although the total RNA was enriched for poly (A) mRNA prior to sequencing and sequences less than 100 bp removed prior to downstream analysis, small sized non-coding RNA had been observed in the pool of unigenes that had neither protein annotation nor potential protein coding sequence. Twenty four unigenes had matched small non-coding RNA and were classified into 6 categories: 5S rRNA (15), 5.8S rRNA (1), RNaseP (3), tRNA (3), U1 (1) and miRNA mir598 (1) (Table S2 in File S2). The unigene predicted to be miRNA mir598 however, did not include the sequence of mature miRNA mir598 and thus has been reclassified as unknown messenger like non-coding RNA. The remaining unigenes that were not classified as any of the non-coding RNA aforementioned will be further filtered and used to mine precursors for novel miRNA genes and described in a separate paper.

### Repetitive element identification and SSR characterization

#### Repetitive elements

Out of 57,891,795 bp of non-ambiguous sequence, 756,405 bp (1.31%) of the transcriptome were identified to contain repetitive elements. Simple repeats were the most abundant repetitive element followed by retroelements and DNA transposons. Specific characterization of each repetitive element is summarized in Table 2.

**Table 2:** The distribution of repetitive elements in *M. rosenbergii* transcriptome.

	Number of elements	Length occupied (bp)	Percentage of sequence (%)
Retroelements	728	76,814	0.13
Penelope	3	165	0.00
LINEs	359	32, 845	0.06
LTR elements	369	43, 969	0.08
DNA transposon	124	13,313	0.02
Unclassified	11	554	0.00
Total interspersed repeats		90,681	0.16
Satellites	6	675	0.00
Simple repeats	8,332	31,8339	0.55

### EST-microsatellite (EST-SSR): mining and validation

It was observed that 7,159 unigenes (2,373 BLAST annotated protein coding genes; 203 predicted protein coding genes; 3 predicted small ncRNA genes; 4,580 others) contained one or more simple sequence repeats (SSR). The repeat motifs in the overall transcriptome can be categorized as follows: 6 dinucleotide repeats, 20 trinucleotide repeats, 47 tetranucleotide-repeats, 80 pentanucleotide repeats and 28 hexanucleotide repeats. The most prevalent SSR observed in the transcriptome were trinucleotides (39.8%) and dinucleotides (33.4%) while the pentanucleotides (2.60%) were the least common.

EST associated microsatellites, also known as EST-SSR or genic microsatellites are most often associated with the coding genes that are conserved across species over large evolutionary time scales. This allows the study of association of these SSR with the function of the genes and the identification of conserved regions in the genomes of different species and genera by using EST-SSR as the anchor marker [30]. Genomic SSR has been identified and characterized in *M. rosembergii* [31], [32], [33], [34], [35]. Up to this date, however, no EST-SSR has been isolated in this organism. Thus, this study represents the first attempt to isolate EST-SSR in *M. rosenbergii*.

Out of 8,332 SSR loci identified, 1,730 primer sets were designed to amplify SSR loci located in unique unigene sequences. 736 of them were derived from known protein coding genes (Table S3 in File S2). The repeat motifs included in this pool of unigenes were dinucleotides (379 loci), trinucleotides (347 loci), tetranucleotides (7 loci) and hexanucleotides (3 loci).

Out of 35 loci selected from 736 loci identified above, PCR validation showed that 23 SSR loci could be amplified successfully, while 12 loci failed to amplify or amplified with larger than expected PCR product size. Failed amplification can be attributed to insertion of an intron in the primer binding region, while larger PCR product size can be caused by insertion of one or more introns in the region close to or within the microsatellite loci. Out of 23 successfully amplified loci, polymorphic screening showed 14 potential polymorphic loci, 4 monomorphic loci and 5 exhibiting null alleles possibly caused by mutation at the primer binding site. Details of the experimental results for successfully amplified loci were shown in Table 1.

### Differential gene expression profiles of different tissues

**Figure 5:**
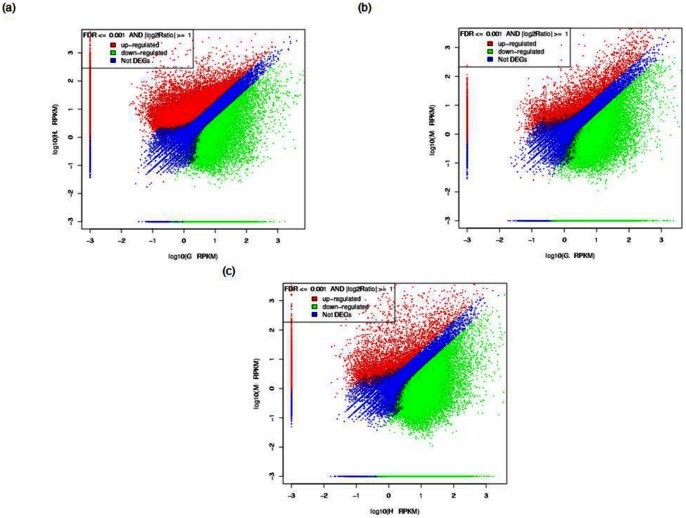
Digital gene expression in two tissues with the following labels: G – Gill, H – Hepatopancreas, M – Healthy Muscle. Each point represents a unigene. The x and y-axis are the log_10_ of the normalized expression level (RPKM) of unigene in the indicated tissue. Red and green points indicate significant change at the absolute value of log_2_ (RPKM ratio in two tissues) ≥1 and FDR  = 0.001. Red points indicate up-regulated unigenes and green points indicate down-regulated unigenes in the tissues in which its expression level is represented by the y-axis. Blue points indicate insignificant differentially expressed unigenes. (a) Expression level of unigenes in G versus H, (b) Expression level of unigenes in G versus M and (c) Expression level of unigenes in H versus M.

The overall differential gene expression profiles of the three tissues (G: gill, H: hepatopancreas; M: normal muscle) were shown in Figure 5 and the number of significant differentially regulated genes in respective tissues was indicated in Table 3 (Table S4 in File S2). The differential gene expression analysis between IHHNV infected and non-infected muscle tissues will be discussed in detail in a separate paper.

**Table 3:** The number of significant differentially regulated unigenes.

Tissue1(t1)	Tissue2(t2)	Differentially regulated unigenes	up-regulated in t1	up-regulated in t2
Hepatopancreas	Gill	50,757	33,157	17,600
Gill	Muscle	38,511	33,297	5,214
Hepatopancreas	Muscle	53,753	48,362	5,391

Hepatopancreas had the most number of up-regulated genes followed by gill and lastly muscle. Hepatopancreas up-regulated genes are almost double of up-regulated genes in gill and nine times the number of up-regulated genes in muscle, while gill had approximately six times the number of up-regulated genes in comparison with muscle. This observation is parallel to the status of hepatopancreas as the most active organ in crustaceans and its multi-functional role analogous to vertebrate's liver and insect's fat body.

#### GO functional enrichment analysis

The results of significant GO enrichment when comparing differentially expressed genes in three tissues are shown in Table 4. Cellular component GO enrichment analysis revealed significant enriched gene expressed in different cellular compartments in all three sets of differential expression analysis (Table 4). GO enrichment in the biological process category for gill and muscle showed significant enrichment in categories related to development.

**Table 4:** The list of Gene Ontology (GO) terms enriched in three tissues and the Bonferroni corrected p-value associated to each enrichment.

Tissue 1	Tissue 2	Enriched GO Categories	
		Category	Corrected p-value
Gill	Hepatopancreas	**Cellular component**	
		myosin II complex	4.22E-02
Gill	Muscle	**Biological process**	
		Developmental process	1.62E-06
		Anatomical structure development	6.69E-05
		Multicellular organismal development	1.00E-04
		Multicellular organismal process	1.06E-03
		Cellular process	1.70E-03
		Cellular component organization	4.81E-03
		System development	8.37E-03
		Vessicle mediated transport	1.87E-02
		**Cellular component**	
		Cytoplasm	4.10E-03
		Actin skeleton	5.74E-03
		Cytoplasmic part	2.00E-02
		Anchoring junction	4.52E-02
Hepatopancreas	Muscle	**Cellular component**	
		Intracellular	1.26E-05
		Intracellular organelle	1.00E-04
		Organelle	1.20E-04
		Intracellular part	2.00E-04
		Cell	3.20E-04
		Cell part	3.20E-04
		Intracellular membrane-bounded organelle	1.09E-03
		Membrane-bounded organelle	1.59E-03

Based on the result, the functional specialization of gill, hepatopancreas and muscle tissues was not captured effectively by the GO annotation system. Since only ∼23% of the protein coding genes in the transcriptome had been annotated using GO system, it is very likely that the shrimp specific protein coding genes are underrepresented. These results were expected since crustacean species have relatively less functional molecular information compared to mammals and insects. However, with the increasing number of high throughput gene expression studies conducted in many crustacean species, the utility of the GO annotation system in functional genomics of non-model animals will definitely improve in future as increasing amounts of crustacean transcriptome data are incorporated into the system.

#### KEGG pathway enrichment analysis

Out of 16,403 unigenes mapped to the KEGG pathways, 9,603 (hepatopancreas and gill), 11,385 (hepatopancreas and muscle) and 9,370 (gill and muscle) unigenes were differentially expressed in two respective tissues. Significance tests showed that 40 pathways were highly enriched when comparing hepatopancreas to gills (File S3), 33 pathways when comparing muscle to gill, and 22 pathways when comparing muscle to hepatopancreas (Table S5 in File S2).

### Reconciling pathway enrichment data with functions

With almost ∼50% (16,403 unigenes) of the protein coding genes represented by the KEGG annotation system, the pathway enrichment was able to capture the functional specialization of the prawns' gill and hepatopancreas tissues compared to GO functional enrichment. This observation can be validated based on known functional roles obtained through extensive mechanistic studies covering various biological aspects in prawns as well as other closely related crustaceans.

The functional diversities of the gills and hepatopancreas were much higher in comparison to the muscle. This is comparable to the proposed multi-functionalities (metabolism, immune and environmental stress response) of hepatopancreas and gill in crustaceans [37], [38]. The number of genes up-regulated in the muscles in comparison to the other two tissues was very small so much so that most of the significantly enriched pathways showed down-regulation of majority of the genes in the muscles. Hepatopancreas and gill on the other hand might share similar function, complement or interact with each other to perform metabolic process in the organism, as active regulations of genes by both tissues can be seen in most of the significantly enriched pathways listed in File S3. Thus, this present study focused on the organ functions of gill and hepatopancreas based on the results obtained from differential gene expression and pathway enrichment analysis of the tissues from these two organs.

#### Metabolism: nutrition

Knowledge on the nutritional requirements of the omnivorous *M. rosenbergii* are essential to determine appropriate feed formulation for optimal survival and growth of the organism that lives in the clear water systems where natural food is not available. *M. rosenbergii* in general requires high carbohydrate and protein for energy and growth, and smaller portions of lipids, vitamins and minerals for proper physiological processes in the organism.

Hepatopancreas is the major organ for metabolism in crustaceans. It plays vital role in the synthesis of digestive enzymes, and in secretion, digestion, nutrient absorption, excretion, reserve (lipid and glycogen) storage and mobilization [36], [37]. These metabolic activities are strongly influenced by physiological demands (molting, reproduction, digestion process, disease) and environmental change (hypoxia) [39].

In the context of nutritional metabolism in hepatopancreas and gill, pathway enrichment showed significant regulation of amino acids, carbohydrates, lipid, glycan, ‘vitamins and cofactors', and ‘terpenoids and polyketides’ metabolism in both tissues (File S3). The metabolism profiles presented in this study have addressed some uncertainties in terms of metabolic capability, nutritional benefits and diversifying roles of digestive enzymes in prawns, as raised by various nutritional studies in *M. rosenbergii* as well as in other crustaceans.

Freshwater prawns require the same ten essential amino acids, EAA (Arginine, Histidine, Isoleucine, Leucine, Lysine, Methionine, Phenylalanine, Threonine, Trytophan and Valine) as other crustaceans and fish species for growth. Evaluation index based on A/E indices (A: proportion of individual EAA; E: proportion of total EAA) has been calculated to assess the amino acid requirement in *M. rosenbergii*. From the ten amino acids, Arginine, Leucine, Lysine and Phenylalanine constitute the highest composition in amino acid profile of *M. rosenbergii, Penaeus monodon* and *Marsupenaeus japonicus* [40]. In this present study, amino acids Phenylalanine, Tyrosine and Histidine metabolisms were significantly enriched in gill and hepatopancreas. Even though Tyrosine cannot be synthesized endogenously by freshwater prawns, it can be synthesized from Phenylalanine when Phenylalanine is sufficiently supplied [41]. It was observed that Phenylalanine conversion to Tyrosine is up-regulated in the gill. Tyrosine is mainly metabolized for the synthesis of acetoacetate in gill; thyroxine and L-DOPA derivatives (e.g. dopamine, noradrenaline) in both hepatopancreas and gill. In gill, Histidine is mainly metabolized into histamine which is an important neuromodulator or neurotransmitter in crustaceans.

Prawns utilize carbohydrate more efficiently compared to protein and lipid for energy especially during starvation [42]. Similar to other marine shrimps and fish, freshwater prawns utilize complex polysaccharides such as starch and dextrin more effectively compared to mono- and di- saccharides [43], [44]. In the present study, starch, dextrin, sucrose, maltose and galactose were highly metabolized to monosaccharide/monosaccharide monophosphate for active energy production and glucuronoside/glucuronate metabolites for excretion in gill and hepatopancreas. Active carbohydrate metabolism observed suggests that both tissues, especially hepatopancreas, play an active role in energy supply in *M. rosenbergii.*

Active regulation of glucosamine (an amino sugar and intermediates of glucose and chitin) metabolism and N-acetylglucosamine/chitin interconversion observed in the two tissues could be attributed to (1) the exogenous chitin digestion which has been shown to exhibit nutritional benefit in shrimps [45] and/or (2) molt cycle which involves chitin degradation of the old procuticle layer and chitin biosynthesis to form new procuticle layer using the building blocks obtained from the degraded endogenous chitin and/or the diet. The chitinase gene exists in multiple isoforms in marine shrimps such as *Fenneropenaeus chinensis, Litopenaeus vannamei* and *Penaeus monodon*. In *P. monodon,* the chitinase isoforms *PmChi-1* and *PmChi-3* were shown to express constitutively in the hepatopancreas tissues and thus they are suggested to be involved in chitin exogenous digestion. However, *PmChi-1* also exhibits a significantly higher expression during premolt in *P. monodon* [46], [47]. In addition, *PmChi-2,* similar to *Pjchi-2,* a molt-related chitinase isoform in *M. japonicus,* is highly expressed in gill especially during intermolt and down-regulated during post-molt. This fluctuating expression pattern during the molt cycle suggests *PmChi-2*′s direct role in molting [47], [48]. Thus, the repertoire of differentially regulated chitinase isoforms in gill and hepatopancreas are expected to be involved in chitin digestion for nutritional purpose, molting or other diversified roles of chitinase in *M. rosenbergii.*

Polyunsaturated fatty acids (PUFA) are suggested to facilitate lipid storage and energy metabolism in the hepatopancreas in *M. rosenbergii* [49]. Linoleic acid (18:2 n-6 PUFA, LNA) has been shown to improve fecundity in female prawns and ammonia stress tolerance in the larvae [50]. In the present study, LNA was extracted from lecithin, a major constituent contributing phospholipids to the freshwater prawn diet, and metabolised into epoxyoctadecenoic acid by cytochrome p450. The freshwater prawn's metabolic ability to convert LNA to arachidonic acid (20:4 n-6 PUFA, ARA) similar to marine shrimps has been proposed in the nutritional study [51]. On the other hand, another study had shown that ARA in freshwater prawn *Macrobrachium borelli* was not synthesized endogenously [52]. The present study showed absence of enzymatic reaction to convert LNA to ARA although ARA (obtained from lecithin) metabolism was present in both tissues. Thus, it is more likely that *M. rosenbergii* ARA body composition is a product of exogenous ARA rather than through endogenous synthesis from shorter chain PUFA.

Carotenoid, the precursor for retinoids in crustaceans, has been established to play roles in pigmentation, as the source of dietary provitamin A, as an antioxidant, as well asbeing involved in sexual maturation, early development and growth. Retinoids, which can be converted to various retinoic acid isomers, is suggested to be involved in embryonic development and cell differentiation [53]. In *M. rosenbergii*, carotenoid supplementation has been documented to result in the reduction of the incidence of Appendage Deformity Syndrome (ADS) physically characterized by poor growth, deformed rostrum and appendages, irregular carapace, cut antennae and low survival [54]. All-trans retinoate treated *M. rosenbergii* embryos and larvae show primordial germ cells increase in the embryo and earlier gonad development in the larvae [55]. The present study showed that β-carotene was the main source of various isomers of retinol, retinal and retinoate metabolites in the tissues. Up-regulation of enzymes in producing all-trans-retinol derivatives was observed exclusively in hepatopancreas while enzymes for retinoic acid synthesis were up-regulated in the gill. Enzymatic system for synthesis of all-trans-retinoate derivatives were actively regulated in both tissues. These observations show that freshwater prawns might have the enzymatic mechanism to metabolize β-carotene into retinoid metabolites. Although the specific function of retinoid metabolites which are differentially synthesized in gill and hepatopancreas is not clear, the physiological function in general might involve activation of hormonal nuclear receptors (Retinoid X receptors, Retinoic Acid Receptor) [53].

#### Steroid hormone biosynthesis and “progesterone-mediated” oocyte maturation

Vertebrate-like steroid hormone synthesis enzymes have been detected in the ovary and hepatopancreas of *Marsupenaeus japonicus* [56] as well as in *M. rosenbergii* [57] Since the hepatopancreas in crustaceans is the equivalent of the liver in vertebrates, it was suggested that this tissue might play a role in catabolism of steroid hormones similar to the vertebrate liver [56]. Using the vertebrate steroid hormone biosynthesis pathway as the reference, the present study showed the possible presence of an enzymatic system involved in steroid hormone biosynthesis for progesterone (3β-hydroxysteroid hydrogenase, 3β-HSD) and androgen (17β-HSD) both of which were mainly up-regulated in hepatopancreas tissue. However, there was no possible presence of endogenous 17α-hydroxylase (conversion of progesterone to 17α-hydroxyprogesterone), C_17_–C_20_ lyase (conversion of 17α-hydroxyprogesterone to androgens) and aromatase activity (conversion of androgen to estrogen) in both gill and hepatopancreas tissues. These results were largely in agreement with the study conducted by Swevers *et al*., (1999) [58] where substantial enzyme activities of HSD but not aromatase were observed in other crustaceans [58]. Summavielle *et al*., (2003) [56] in their study suggested that progesterone conversion in the shrimp hepatopancreas might be activated by a different bioconversion pathway [56].

Effects of the vertebrate-like steroid hormones on reproductive process such as oocyte maturation in crustaceans still remain unresolved. In *M*. *rosenbergii*, hepatopancreas plays a vital role in reproduction, as it has been reported to be the principal extra-ovarian synthesis site for vitellogenin (Vg), which is the yolk precursor of an important nutrient for developing embryos – vitellin [59], [60], [61].

The extraovarian Vg, which has been cleaved into two subunits in the hepatopancreas, is transported through the haemolymph to the developing oocyte before it is sequestered and processed into vitellin by the ovary to induce oocyte maturation [62]. In *M. rosenbergii,* two previous studies have shown fluctuations of 17β-estradiol and progesterone during reproductive molt cycle but 17β-estradiol remained basal and progesterone was absent during non-reproductive molt cycles respectively [63], [64].

In the present study, KEGG's progesterone-mediated oocyte maturation pathways were significantly enriched in the differential gene expression analysis between hepatopancreas and two other tissues. In the pathway enrichment, most genes downstream of the translation of maternal mRNAs were up-regulated in the hepatopancreas, indicating a major role of this tissue in oocyte maturation. In vertebrates, the regulation of oocyte maturation is mediated by innumerable intracellular signals. cAMP maintains meiotic arrest while translation of the MOS gene, which is regulated by Hsp90, activates the MAPK cascade, cyclin-dependent kinase 1 and the positive feedback loop to trigger maturation [65].

Several factors, namely cytoplasmic polyadenylation element binding protein (CPEB), Hsp90 and suppression of cAMP/PKA signaling were shown to initiate maturation. High Hsp90 relative gene expression in hepatopancreas was consistent with the observation achieved by Wu and Chu (2008) [66] in which the Hsp90 expression in hepatopanceas was found to be higher than in gill especially during ovarian maturation. In addition, estrogen level and heat shock protein 90 (Hsp90) gene expressions in the ovary and hepatopancreas strongly correlates during ovarian development in *Metapenaeus ensis* [66]. These observations suggest a similarity, to a certain extent, in the mechanism of vitellogenesis regulation between the vertebrates and crustaceans. However, since the presence of steroid hormone receptors in crustaceans is still in dispute, the vertebrate-like steroid hormone mediated oocyte maturation in *M. rosenbergii* remains as an open question.

#### Infectious disease from Vibrio and innate immune system

Gills in crustaceans are vital organs that play important roles in respiration, regulation of osmotic and ionic balance, detoxification, excretion and immune defense against microbial pathogens which is demonstrated by a marked increase of haemocytes in the gill during infection [67], [68]. A previous study also showed that the gene expression in haemocytes and gills are quite similar, suggesting that the circulating haemocytes are infiltrating the shrimp gill tissue [69]. Aside from hepatopancreas and lymphoid organs, gills were also shown to be the main accumulation site for intact and/or degraded products of bacteria such as *Vibrio.* It was proposed that gills trap the bacterial-haemocyte nodules when they reach a certain size before phagocytosis occurs [68]. Larger accumulation of *Vibrio* in gills was observed in gills under the oxygen stress (hypoxia) possibly because hypoxia can cause the suppression of the immune defense mechanism of the haemocytes [70]. In contrast, a study conducted in crab showed that immune response in gill would decrease the efficiency of gill in gas exchange that may lead to suffocation and death in the organism [71].

Pathways enriched for the differential study involving gills were mainly categorized in infectious disease, signaling transduction, as well as in the immune system (Table S5 in File S2). This can be attributed to the gills' direct contact with biotic and abiotic factors in the external environment that requires constant detection (or recognition) and signaling transduction to mediate the response of the epithelial cells lining the gills against these external factors. Biotic factors or opportunistic pathogens and parasites (e.g. Gram negative bacteria within the genera *Vibrio*, filamentous bacteria, protozoan and fungi) are commonly found in the freshwater culture system in the hatcheries. With the high exposure to these opportunistic pathogens, Crustaceans in general respond by releasing pattern recognition proteins, antimicrobial peptides, clotting proteins, and prophenoloxidase as part of its immune defense against pathogens and parasites. Selected genes such as antimicrobial peptide, propenoloxidase nad recognition proteins were analyzed functionally using real-time PCR expression. (Arockriaj et al., 2011 and 2012).

#### Xenobiotics metabolism, oxidative stress and ‘replication and repair

Gills and hepatopancreas have been the target organs for the study of environmental stress, that has mainly focused on heavy metals and xenobiotics toxicity in aquatic crustaceans, because gill is in direct contact with and accumulates pollutants in the water in the mitochondria and because hepatopancreas is the main organ for accumulation and detoxification of these compound in the lysosomes [71], [72], [73], [74], [75]. The R cells in hepatopancreas in particular have the ability to perform biotransformation using enzymes such as cytochrome p450, to sequester and detoxify xenobiotics [76]. Heavy metal and xenobiotic toxicity in aquatic organisms is characterized by mitochondrial dysfunction, oxidative stress, and DNA repair impairment which are also the hallmark of neurodegenerative diseases such as amyotrophic lateral sclerosis, Parkinson and Huntington disease in the vertebrates [77], [78]. Oxidative stress induced by either high exposure to reactive oxygen species (ROS) or decreased antioxidant defense by antioxidant enzymes such as metallothioneins, sodium dismutase and glutathione-S-transferase [79] can cause lipid peroxidation, cell injuries or death, disruption of calcium homeostasis, and genotoxicity (damage to the genetic materials) [80], [81], [82]. Since genotoxicants can cause irreversible effects, namely transcriptional errors, mutagenesis and cell death, the exposed organisms need efficient mechanisms to repair the DNA molecules to reverse the damage [83], [84].

The responses of gill and hepatopancreas tissues to environmental toxicity were very well represented in the biological pathway enrichment in our study where pathways involving ‘xenobiotics biodegradation and metabolism’, ‘neurodegenerative diseases’, ‘transport and catabolism’, as well as ‘replication and repair’ were significantly enriched in the differential gene study involving these two tissues. High gene expression of hepatopancreas in the context of ‘replication and repair’ is consistent with the experimental observation of the effects of cadmium to the repair systems in gills and hepatopancreas in marine crabs (*Charybdis japonica)*, where significant drops in the ratio of intact double stranded DNA to total DNA in gill were observed in comparison to insignificant changes in the hepatopancreas [82]. Both the experimental data and the molecular study here showed that hepatopancreas had higher capability to perform DNA repair to cope with the damage caused by environmental genotoxicants.

Even though it was not designed to specifically address toxicological aspects of *M. rosenbergii*, this study has demonstrated the possible utility of large scale gene expression studies using RNA-seq to address research questions by taking advantage of the shared features between vertebrates and crustaceans. Although several transcriptome profiling studies using DNA microarray technology for ecotoxicogenomics have been attempted in *Daphia magna* [85], [86], the novel discoveries were limited to the availability of the sequence data of the organism of interest. Therefore the results, especially the molecular data, from this study provide the opportunity to pursue further research in *M. rosenbergii* ecotoxicogenomics [87]–[92].

## Conclusions

The present study represents a step forward in identifying a number of possible conserved genes that are likely to be involved in various important biological activities by providing an extensive catalogue of 102,323 expressed genes from freshwater shrimp. Analyzing the prawn transcriptome data through homology search and modeling based methods GO and KEGG pathway has led to the recapitulation of various experimental studies done on prawns or crustaceans and revelation of possible shared function with model organisms that have been well studied. With the rapid advance of experimental annotation of functional genomic data for organisms with available genome data, the utility of this method is still expanding. However, careful selection of the model based representations of the transcriptome utilizing current knowledge from model vertebrates is needed. Thus validation of any novel discovery experimentally need to be done to avoid misinterpretation of the functional genomics data. This is because of the extensive divergence of some genes and biological pathways between the model vertebrates and invertebrates since their most recent common ancestor. The functional genomics resource generated from this study provides the basis for generating hypotheses to guide future molecular research in freshwater shrimp as well as for the assembly and annotation of the *Macrobrachium rosenbergii* genome.

## Supporting Information

File S1 **Table** **S1,** NCBI Nr, Swissprot, KEGG, COG and GO annotation. The annotation from the BLAST results of unigenes with significant hits (E-value ≤ 10^−5^) to known protein coding genes in Nr, Swissprot, KEGG and COG databases are provided. Unigenes with positive BLAST results were further classified according to its associated GO terms. **Table** **S2,** Raw and normalized expression value of unigenes in three libraries (Gill, Hepatopancreas and Healthy Muscle). Raw expression value of each unigene represented by number of clean reads from each library successfully mapped to the corresponding unigene. Normalized expression value was represented by the RPKM value. The length of each unigene was also included.
(XLS)

File S2 **Table** **S1,** Novel protein coding genes predicted by ESTScan. The coding sequence start site, end site, length and strands are provided. **Table S2**, Non-coding RNA annotation. Non-coding RNA annotation of the unigenes is performed using INFERNAL cmsearch. The matched start and end site, E-value, score, GC content, Rfam accession number and ID from the results are provided. **Table S3**, Primer sets designed to flank EST-SSR that is associated with annotated (known) protein coding genes. **Table S4**, Significant differentially expressed genes between two libraries. Log_2_ of the ratio of normalized expression value of unigene, up or down regulation, p-value and FDR value are provided. **Table** **S5,** KEGG pathway enrichment results. Number of differentially regulated unigenes and total number of unigenes in the transcriptome mapped to each pathway, hypergeometic p-value, FDR and the pathway ID are provided.
(XLS)

File S3 **Results of significantly enriched KEGG pathways for differential expression study comparing gill and hepatopancreas.**
(DOC)
