# Supplementary material for: In-Depth Tanscriptomic Analysis on Giant Freshwater Prawns
Source: PLoS One. 2013 May 29;8(5):e60839. doi: 10.1371/journal.pone.0060839 (PMC3667022; doi:10.1371/journal.pone.0060839)
Supplement: File S3 — Results of significantly enriched KEGG pathways for differential expression study comparing gill and hepatopancreas. (DOC) [file pone.0060839.s003.doc]

**Figure S1:Results of significantly enriched KEGG pathways for differential expression study comparing gill and hepatopancreas.**

| Category | Pathway-general | Pathway-specific | Diff: 9,603  All: 16,403 | Pvalue | Qvalue |
| --- | --- | --- | --- | --- | --- |
| Cellular processes | Cell growth and death | Cell cycle | 260(400) | 4.38E-03 | 2.65E-02 |
|  | Transport and catabolism | Lysosome | 278(409) | 4.45E-05 | 4.85E-04 |
| Environmental information processing | Signaling molecules and interaction | Neuroactive ligand-receptor interaction | 180(222) | 5.19E-13 | 1.13E-10 |
|  |  | ECM-receptor interaction | 207(281) | 7.15E-08 | 2.84E-06 |
|  |  | Cytokine-cytokine receptor interaction | 101(145) | 3.65E-03 | 2.28E-02 |
| Genetic information processing | Replication and repair | DNA replication | 82(98) | 7.81E-08 | 2.84E-06 |
|  |  | Mismatch repair | 61(69) | 5.51E-08 | 2.84E-06 |
|  |  | Base excision repair | 83(103) | 1.62E-06 | 3.21E-05 |
|  |  | Nucleotide excision repair | 82(105) | 1.85E-05 | 2.51E-04 |
|  |  | Homologous recombinantion | 49(59) | 5.09E-05 | 5.28E-04 |
|  | Translation | Aminoacyl-tRNA biosynthesis | 68(93) | 2.44E-03 | 1.56E-02 |
| Human diseases | Cardiovascular diseases | Hypertrophic cardiomyopathy (HCM) | 289(408) | 1.31E-07 | 3.57E-06 |
|  |  | Dilated cardiomyopathy | 288(418) | 6.27E-06 | 9.77E-05 |
|  | Infectious diseases | Amoebiasis | 351(516) | 4.20E-06 | 7.04E-05 |
|  |  | Vibrio cholerae infection | 311(470) | 3.49E-04 | 2.82E-03 |
|  | Neurodegenerative diseases | Prion diseases | 113(158) | 4.69E-04 | 3.41E-03 |
|  |  | Amyotrophic lateral sclerosis (ALS) | 210(316) | 2.17E-03 | 1.43E-02 |
| Metabolism | Amino acid metabolism | Tyrosine metabolism | 94(112) | 6.31E-09 | 4.59E-07 |
|  |  | Phenylalanine metabolism | 45(56) | 4.61E-04 | 3.41E-03 |
|  |  | Histidine metabolism | 34(42) | 1.82E-03 | 1.24E-02 |
|  | Carbohydrate metabolism | Galactose metabolism | 67(81) | 2.83E-06 | 5.13E-05 |
|  |  | Starch and sugar metabolism | 97(128) | 3.15E-05 | 3.94E-04 |
|  |  | Pentose and glucuronate interconversions | 56(71) | 2.42E-04 | 2.11E-03 |
|  |  | Amino sugar and nucleotide sugar metabolism | 110(152) | 2.64E-04 | 2.22E-03 |
|  | Glycan biosynthesis and metabolsim | Glycosaminoglycan degradation | 73(97) | 4.22E-04 | 3.29E-03 |
|  |  | Other glycan degradation | 57(78) | 5.42E-03 | 3.11E-02 |
|  |  | Glycosphingolipid biosynthesis-globo series | 38(50) | 7.62E-03 | 4.15E-02 |
|  | Lipid metabolism | Steroid hormone biosynthesis | 76(92) | 6.73E-07 | 1.63E-05 |
|  |  | Linoleic acid metabolism | 63(87) | 5.04E-03 | 2.97E-02 |
|  | Metabolism of cofactors and vitamins | Retinol metabolism | 101(125) | 9.41E-08 | 2.93E-06 |
|  |  | Ubiquinone and other terpenoid-quinone biosynthesis | 32(37) | 2.40E-04 | 2.11E-03 |
|  | Metabolism of terpenoids and polyketides | Limonene and pinene degradation | 57(70) | 3.94E-05 | 4.52E-04 |
|  | Xenobiotics | Metabolism of xenobiotics by cytochrome P450 | 90(113) | 1.57E-06 | 3.21E-05 |
|  | Biodegradation and metabolism | Drug-metabolism-other enzymes | 103(137) | 3.25E-05 | 3.94E-04 |
|  |  | Drug-metabolism-cyctochrome P450 | 85(112) | 8.74E-05 | 8.66E-04 |
| Organismal systems | Development | Dorsal-ventral axis formation | 287(269) | 1.17E-04 | 1.11E-03 |
|  | Endocrine system | Renin-angiotensin system | 45(57) | 9.48E-04 | 6.67E-03 |
|  |  | Progesterone-mediated oocyte maturation | 183(277) | 5.81E-03 | 3.25E-02 |
|  | Immune systems | Complement and coagulation cascades | 148(187) | 1.68E-09 | 1.84E-07 |
|  |  | Hematopoietic cell lineage | 98(128) | 1.36E-05 | 1.97E-04 |
